# Evaluation of Seven Different RNA-Seq Alignment Tools Based on Experimental Data from the Model Plant *Arabidopsis thaliana*

**DOI:** 10.3390/ijms21051720

**Published:** 2020-03-03

**Authors:** Stephanie Schaarschmidt, Axel Fischer, Ellen Zuther, Dirk K. Hincha

**Affiliations:** Max Planck Institute of Molecular Plant Physiology, Am Mühlenberg 1, 14476 Potsdam, Germany; schaarschmidt@mpimp-golm.mpg.de (S.S.); afischer@mpimp-golm.mpg.de (A.F.); zuther@mpimp-golm.mpg.de (E.Z.)

**Keywords:** *Arabidopsis thaliana*, differential gene expression, natural genetic variation, read mapping tools, RNA-Seq

## Abstract

Quantification of gene expression is crucial to connect genome sequences with phenotypic and physiological data. RNA-Sequencing (RNA-Seq) has taken a prominent role in the study of transcriptomic reactions of plants to various environmental and genetic perturbations. However, comparative tests of different tools for RNA-Seq read mapping and quantification have been mainly performed on data from animals or humans, which necessarily neglect, for example, the large genetic variability among natural accessions within plant species. Here, we compared seven computational tools for their ability to map and quantify Illumina single-end reads from the *Arabidopsis thaliana* accessions Columbia-0 (Col-0) and N14. Between 92.4% and 99.5% of all reads were mapped to the reference genome or transcriptome and the raw count distributions obtained from the different mappers were highly correlated. Using the software DESeq2 to determine differential gene expression (DGE) between plants exposed to 20 °C or 4 °C from these read counts showed a large pairwise overlap between the mappers. Interestingly, when the commercial CLC software was used with its own DGE module instead of DESeq2, strongly diverging results were obtained. All tested mappers provided highly similar results for mapping Illumina reads of two polymorphic Arabidopsis accessions to the reference genome or transcriptome and for the determination of DGE when the same software was used for processing.

## 1. Introduction

Since the completion of the human genome project in 2003 [1], sequencing technologies have developed extraordinarily fast. The resulting data have revealed the astonishing complexity of genome architecture and transcriptome composition. In this context, transcript identification and the quantification of gene expression play crucial roles in connecting genomic information with phenotypic and biochemical measurements. These two key aspects of transcriptomics can be combined in a single high-throughput sequencing assay called RNA-Sequencing (RNA-Seq). This approach allows detailed transcript profiling including the identification of splicing-induced isoforms, nucleotide variation and post-transcriptional base modification [2].

While comparative studies of diverse read aligners have been performed using data with a corresponding reference genome or transcriptome [3,4,5,6,7] or *de novo* assembly [8,9,10], only little evaluation is available of the performance of read mappers for data generated from genotypes within a species showing sequence polymorphisms. In this study, the algorithmically different mappers bwa, CLC Genomics Workbench, HISAT2, kallisto, RSEM, salmon and STAR were used to map experimentally generated RNA-Seq data from the two natural accessions Columbia-0 (Col-0) and N14 of the higher plant *Arabidopsis thaliana* and to quantify the transcripts.

Bwa (Burrows–Wheeler-Alignment) was developed for mapping short DNA sequences against a reference genome and was extended for RNA-Seq data analysis. For indexing, the algorithm constructs a suffix array and Burrows–Wheeler-Transformation (BWT), and subsequently matches the sequences using a backward search [11]. STAR (Spliced Transcripts Alignment to a Reference) is a specialized tool for RNA-Seq reads that uses a seed-extension search based on compressed suffix arrays [12] and can detect splice-junctions. HISAT2 (Hierarchical Indexing for Spliced Alignment of Transcripts 2) is also a splice-aware aligner using a graph-based alignment approach (graph Ferragina Manzini index) that can align DNA and RNA sequences [13]. RSEM (RNA-Seq by Expectation Maximization) is a software package that quantifies transcript abundances. It can employ different pre-defined mappers such as bowtie2 and based on the generated alignments utilizes a maximum likelihood abundance estimation, the expectation-maximization algorithm, as the statistical model to quantify transcripts [14]. By contrast, salmon and kallisto are tools which do not perform a classical alignment of individual bases, but instead implement new strategies for RNA-Seq quantification. Salmon is based on the concept of quasi-mapping. It uses a suffix array that is BWT-indexed and searched by an FMD algorithm, allowing the discovery of shared substrings of any length between a read and the complete set of transcripts. Mismatches are handled with chains of maximally exact matches [15]. The concept of kallisto is based on *pseudo-alignments*. Pseudo-alignments define a relationship between a read and a set of compatible transcripts. This relationship is computed based on “mapping” the *k*-mers to paths in a transcript De Bruijn graph. As the pseudo-alignments are generated, equivalence classes are computed and used for the relative isoform quantification [16]. CLC read mapping utilizes an approach described by Mortazavi et al. [3] and is the only commercial tool with a graphical user interface included in our study. 

**Figure 1:**
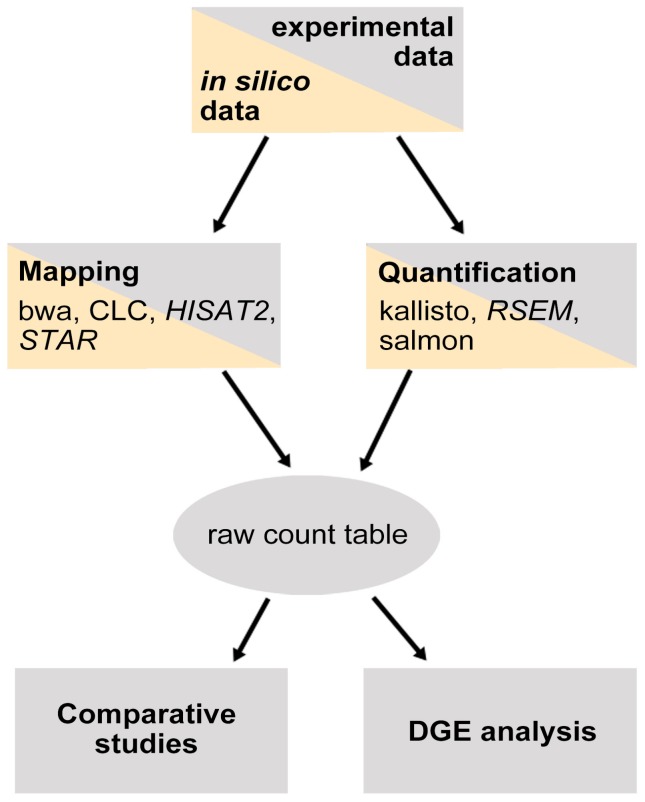
Analysis workflow. Light gray represents all steps performed for experimental data, light orange for analysis of in silico generated data analyzed with HISAT2, RSEM and STAR.

Here, we compare the performance of these seven RNA-Seq mappers in the analysis of experimentally generated transcriptome data covering more than 30,000 *Arabidopsis thaliana* genes. The analysis compares alignment accuracy and quantification to enable comprehensive biological interpretation. For the RNA-Seq experiment, RNA was isolated from the higher plant *Arabidopsis thaliana* and the performance of each software was tested on 150 bp single-end reads from the two natural accessions Col-0 and N14 [17]. Mappability, raw count expression, overall similarity of the count distribution and differential gene expression (DGE) were analyzed to compare the mappers. The two splice-aware aligners HISAT2 and STAR were compared for accuracy by mapping the reads against the reference genome without an annotation. Additionally, an in silico approach to characterize the correctness of the mappers was performed (see Figure 1 for a schematic description of the analysis workflow). 

## 2. Results

### 2.1. Mapping Statistics

**Figure 2:**
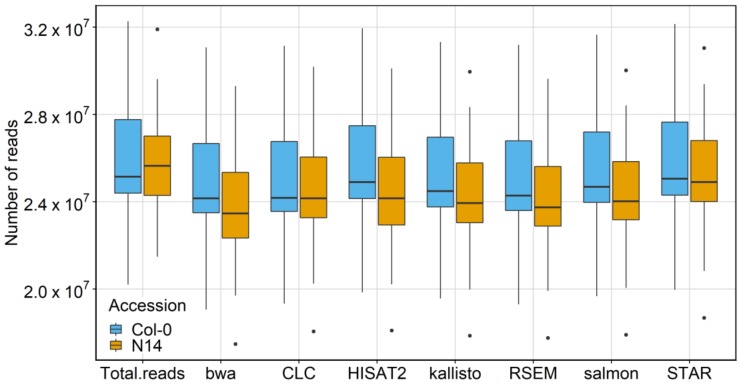
Mapper comparison based on mappability. Number of mapped reads against the Col-0 reference sequence for all seven mappers and each accession separately. The analysis included RNA-Seq data from 36 biological samples. Outliers for N14 were in each case sample V for minimum, sample AF for maximum (see Table A3 for sample information).

After pre-processing, the resulting dataset contained 36 samples [17] with a sequencing data size ranging from about 21 to almost 33 × 10^6^ reads (Table A1). In general, a high fraction of the total reads was mapped for both accessions. The mapping for Col-0 was slightly better than for N14 (Figure 2) with mapped reads between 95.9% (bwa) and 99.5% (STAR). For N14 between 92.4% (bwa) and 98.1% (STAR) of the reads were mapped against the respective reference sequence of Col-0 (Table A2). 

### 2.2. Raw Count Distribution for Individual Samples

**Figure 3:**
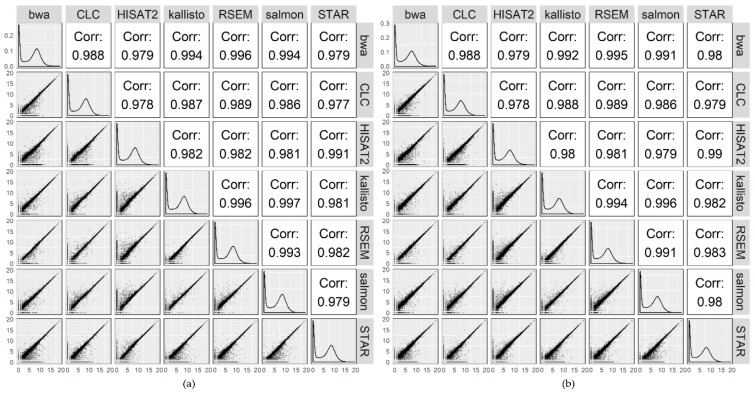
Raw counts of mapped reads determined by each mapper plotted against each other. Results are shown for sample A of Col-0 (**a**) and sample B of N14 (**b**) which both were obtained from plants grown under control conditions at 20 °C (see Table A3 for sample information). Lower triangle represents scatterplots of log_2_(counts + 1) transformed, unfiltered raw counts for each mapper plotted against each other. The diagonal histograms show the density of the raw count distribution for each mapper. The upper triangle displays the correlation coefficients.

Raw count distributions between the mappers were investigated for both accessions. The unfiltered expression values for each mapper were plotted against each other and correlations computed. The results for one control sample of Col-0 (sample A) and N14 (sample B) are shown as an example (Figure 3). For Col-0 (Figure 3a), high correlation coefficients between 0.977 (STAR vs. CLC) and 0.997 (kallisto vs. salmon) were determined. For N14 (Figure 3b) the correlation coefficients ranged from 0.978 (CLC vs. HISAT2) to 0.996 (kallisto vs. salmon). Regarding the STAR and HISAT2 comparisons with all other mappers, a higher variance was observed in the direction of STAR and HISAT2 for lowly expressed genes. 

### 2.3. Overall Comparison of the Mappers

**Figure 4:**
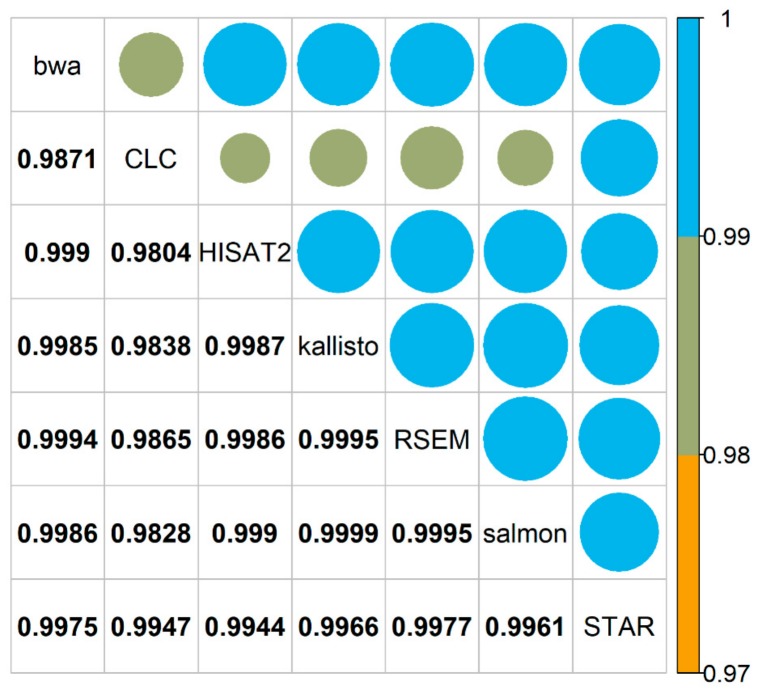
Mapper comparison based on raw count distributions. Graphical representation of the computed R_v_ values based on the correlation matrices of the unfiltered raw count tables generated by all mappers for all samples from both accessions. Values close to 1 indicate high similarity. The color and shape scales were adjusted to visualize the small differences between the R_v_ coefficients.

For a more quantitative comparison, the raw counts generated by each mapper from all samples were compared against each other employing the R_v_ coefficient to quantify similarity. The raw count tables generated by the seven mappers have a high similarity indicated by R_v_ values close to 1 (Figure 4). Salmon and kallisto showed the highest similarity (R_v_ = 0.9999). CLC mapped slightly differently compared to bwa, HISAT2, kallisto, RSEM and salmon. However, it should be stressed that the raw count tables of all mappers were very similar; with 0.9804 as the lowest R_v_ value (CLC vs. HISAT2).

To investigate the effect of mapper choice on further statistical analysis, differentially expressed genes between control and cold acclimated conditions were determined [17]. In the read mapping steps, the aligners bwa, salmon and kallisto, using the transcriptomic reference, identified 32,243 expressed genes and thus 1,359 genes less than the other mappers with 33,602 genes each. This difference is due to the presence of non-coding RNAs such as transfer RNAs (tRNA) and micro RNAs (miRNA) in the genomic reference, which are absent from the transcriptomic reference that is based on poly-adenylated mRNAs. Prior to DGE analysis, transcript raw count tables were filtered to remove lowly expressed genes with less than five counts over all 36 samples, resulting in 23,903 (CLC) to 25,144 (RSEM) genes (Table 1). While this cut-off is admittedly arbitrary, most genes are removed with a cut-off of 1 read count (around 20%), while additional increases from 2 to 10 counts only reduce the number of genes by 2–0.3% per additional count, making the exact cut-off rather uncritical.

**Figure 5:**
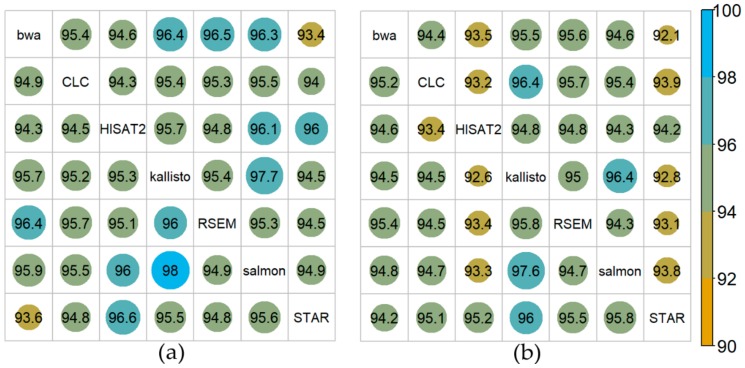
Overlap of significantly differentially expressed genes among the different mappers for cold acclimated vs control plants. Overlap in % for Col-0 (**a**) and N14 (**b**). DGE was determined at FDR *p* < 0.1 and an absolute log_2_FC > 1 using the R-package *DESeq2*. Overlap of differentially expressed genes among each pair of mappers is represented in an asymmetric matrix.

The percentage of overlapping DGE (control vs. cold acclimated) identified by each pair of mappers was analyzed in both directions using *DESeq2* [18] in all cases and was plotted in an asymmetric matrix. For Col-0 (Figure 5a) kallisto and salmon yielded a large overlap of DGE of 98% (kallisto vs. salmon) and 97.7% (salmon vs. kallisto). For N14 (Figure 5b) slightly smaller overlaps were detected, but also here salmon and kallisto (97.6% and 96.4%) yielded the largest overlap. On the other hand, for both Col-0 and N14 the lowest overlaps were detected for bwa and STAR (93.4% and 92.1%, respectively). In general, a smaller overlap of DGE between 92% and 94% was identified for the comparisons of STAR and HISAT2 with the remaining five mappers.

DGE analysis [19,20] was additionally performed directly in the CLC software instead of using *DESeq2*. Using the standard significance levels for these two software packages (FDR < 0.1 and FDR < 0.05 for *DESeq2* and CLC, respectively) this resulted in a much higher number of significantly differentially expressed genes for the two exemplary comparisons, detailed under Methods, compared to the *DESeq2* analysis (Table 2). Also, there was only a limited overlap between the results of the two methods.

All mappers have different options to perform RNA-Seq quantification (Table 3). While most mappers can only use either a genome or a transcriptome reference, CLC, HISAT2 and STAR are able to use both types of reference sequences to align transcripts. Depending on the downstream analysis, it is essential which output each mapper provides. The classical alignment-based mappers bwa, CLC, HISAT2, RSEM and STAR provide an alignment output of the reads against the references, whereas salmon and kallisto only provide read quantifications. Nevertheless, kallisto offers a “pseudo-alignment” which can generate alignment files and salmon provides an option to re-quantify RNA-Seq reads using previously generated alignments against the transcriptome as obtained, for example, from STAR. Five out of the seven mappers generate transcript count tables. Only for HISAT2 and bwa additional tools have to be employed for this purpose.

**Figure 6:**
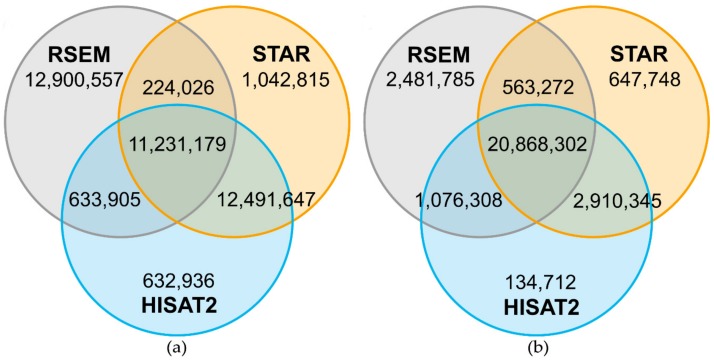
Number of reads mapping on the same genomic position comparing HISAT2, RSEM and STAR for Col-0. Venn diagrams are based on 24,989,667 reads mapped by all three mappers and represent the overlap of mapped reads on the same genomic position for sample A (see Table A3 for sample information). A high number of the uniquely mapped reads in RSEM was based on soft-clipping by one bp performed by HISAT2 and STAR (**a**). The reads in HISAT2 and STAR were corrected by adding the soft-clipped bp back and the overlap with RSEM increased strongly (**b**).

**Figure 7:**
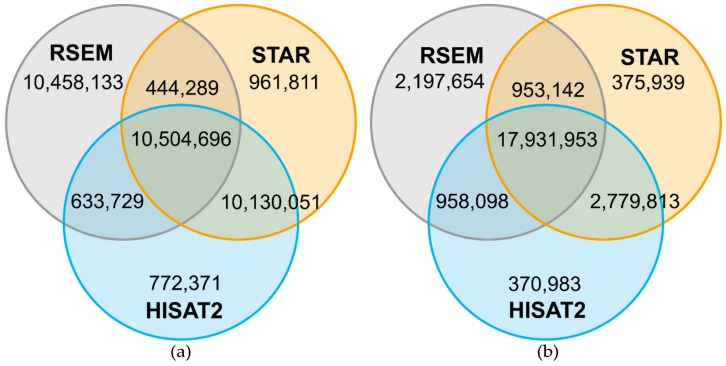
Number of reads mapping on the same genomic position comparing HISAT2, RSEM and STAR for N14. Venn diagrams are based on 22,040,847 reads mapped by all three mappers and represent the overlap of mapped reads on the same genomic position for sample B (see Table A3 for sample information). A high number of the uniquely mapped reads in RSEM was based on soft-clipping by one bp performed by HISAT2 and STAR (**a**). The reads in HISAT2 and STAR were corrected by adding the soft-clipped bp back and the overlap with RSEM increased strongly (**b**).

For a more detailed investigation of the comparability of the outputs of different mappers, three of the seven mappers were analyzed in detail regarding read position on the reference sequence. The overlap of reads from one sample, which were mapped by HISAT2, bowtie2/RSEM and STAR, was determined and the positions of the mapped reads on the reference genome were compared. For Col-0 around 11.2 × 10^6^ (Figure 6a) of around 24.9 × 10^6^ mapped reads and for N14 around 10.5 × 10^6^ reads (Figure 7a) of around 22.0 × 10^6^ mapped reads were located on the same genomic position by all three mappers. For both accessions, bowtie2/RSEM showed a high number of reads mapping to a different position compared to HISAT2 and STAR. The number of reads with a unique position was between 20.4-fold and 10.9-fold higher for bowtie2/RSEM than for the other two mappers. Hence, the differences in read positions were determined, showing that most of these reads had a position that differed by one base pair. This is the result of soft clipping of the first or last base pair that is performed by HISAT2 and STAR. After adding the base pair back to the reads in HISAT2 and STAR, the overlap with RSEM increased to 20.8 × 10^6^ reads for Col-0 (Figure 6b) and to 17.9 × 10^6^ reads for N14 (Figure 7b). However, RSEM still produced between 18.4-fold and 3.8-fold more uniquely positioned reads than HISAT2 and STAR that cannot be explained by soft clipping.

Additionally, the two splice-aware aligners HISAT2 and STAR were tested for accuracy. Reads of all 36 biological samples were mapped against the reference genome sequence without annotation and reads on exons were determined with featureCounts (Table 4). For Col-0, 93% (STAR) and 94% (HISAT2), and for N14 around 91% (both mappers) of the primary alignments were mapped to known exons. A small fraction of reads were not assigned to the annotated exons due to no mapping, multimapping (i.e., mapping to more than one location) or mapping to intergenic regions.

### 2.4. Mapping of in Silico Generated Reads

To investigate whether mappers placed the mapped reads in the correct positions on the reference genome, the alignment results for in silico generated Col-0 RNA-Seq reads were analyzed using HISAT2, bowtie2/RSEM and STAR. All three mappers correctly positioned a high percentage (almost 99%) of the reads on the respective reference sequence (Table 5) for the primary alignments. Almost all remaining reads were mapped to the correct gene, but to a different transcript. Furthermore, only 0.001 to 0.03% of the reads were not mapped against the reference sequence for all mappers. A small number of reads mapped to intergenic regions for STAR and HISAT2 while for bowtie2/RSEM all reads were mapped on known genes. This derives from the fact that the used mapper bowtie2 is a splice unaware aligner that only maps against the transcriptome which was extracted from the genome reference. For the secondary alignments of HISAT2 and STAR, which only constituted 3.2% (STAR) and 3.8% (HISAT2) of the total alignments, 41.5% (HISAT2) and 36.9% (STAR) of the reads were correctly aligned. The majority of the secondary alignments, 55% for HISAT2 and 59% for STAR, mapped the reads to wrong positions, mostly to wrong (unrelated) or paralogous genes. For bowtie2/RSEM, almost 43% of these reads were mapped multiple times. Nearly 96% of these reads were mapped to the wrong gene.

For a better overview, the alignments were split into primary and secondary alignments. If a read maps multiple times against the reference, one mapping is defined as primary (underlying criteria depend on the mapper), while the other mappings are classified as secondary alignments.

## 3. Discussion

RNA-Seq data from the *Arabidopsis thaliana* accessions Col-0 and N14 were mapped with five alignment-based and two pseudo-alignment tools. For Col-0, high mappability of the 150 bp single-end Illumina reads to the Col-0 reference genome or transcriptome was found for all seven alignment tools, ranging from 95.9% (bwa) to 99.5% (STAR). A slightly smaller fraction of the reads obtained from N14 was mapped to the same references, ranging from 92.4% to 98.1%. The high quality of the reference sequences may contribute to the high fraction of mapped reads. For both accessions, bwa had the lowest performance and STAR the highest, although it should be stressed that differences in mappability for any sample between the mapping tools ranged only from 1% to 4%. Comparable performance of different mapping tools has been found in previous studies using either simulated reads or RNA-Seq reads obtained from various non-plant organisms [21,22,23,24,25]. On the other hand, another report showed that seed-extended approaches used by STAR performed better than e.g., exon-first approaches, when mapping reads from genetically polymorphic species [26].

Considering the two accessions separately, the high number of mapped reads for Col-0 is in agreement with the fact that the Col-0 reference sequences were used for mapping. However, a small number of reads was not mapped, potentially due to sequencing errors or to polymorphisms between the publicly available genome sequence and the genome of the Col-0 population used in our experiments. In this context it has to be kept in mind that the Col-0 populations used in various laboratories around the world have been separated for many generations and have very likely accumulated different mutations over time [27]. The generally lower percentage of mapped reads for N14 can be explained by natural variation between the accessions [28,29].

In addition to the percentage of mapped reads, the correctness of the mapping of reads to the reference genome or transcriptome is also of crucial importance to obtain reliable biological information from an RNA-Seq experiment. We found that HISAT2 and STAR had a high overlap of reads mapping to the same position in the reference sequence. The differences in read positions between bowtie2/RSEM and HISAT2/STAR originated to a large part from the soft-clipping, mostly of the first base of the reads, by both aligners. Soft-clipping can be turned off in both tools and that largely eliminates the observed differences. However, STAR has a higher tolerance for more soft-clipped and mismatched bases compared to HISAT2, which leads to a higher mapping rate for STAR and more unmapped reads for HISAT2 [24]. Also, in our analysis, STAR showed the highest fraction of mapped reads for both accessions among all compared mapping tools.

Our analysis of an in silico generated RNA-Seq data set also indicated that differences in the mapping quality between the three mappers are most likely due to their different ability to deal with mismatches. About 99% of the primary aligned reads were correctly positioned and the mappers showed the same performance when synthetic reads without any mismatches between read and reference sequences were used. This indicates that mapper performance may also depend on other factors, such as the complexity of the genome, read length and read quality [22]. The high fraction of correctly mapped reads may in part be due to the comparatively small genome of Arabidopsis with roughly 130 megabases and a low content of repetitive DNA sequences [30,31]. Regarding the secondary alignments, RSEM showed a high number of multimapped reads. The mapping for RSEM was performed with the mapper bowtie2 which searches for distinct, valid alignments for each read. As long as no upper limit is defined, bowtie2 will continue to look for all alignments that are as good or better for one read [32]. If the same read maps multiple times with the same quality string, the primary alignment is chosen randomly. The quantification algorithm of RSEM also depends on a high number of multi-mapped reads.

From a biological point of view, the quantification of gene expression is the most important part of an RNA-Seq experiment as researchers are mostly interested in the identification of differentially expressed genes, either between conditions or between genotypes. Correct mapping, as discussed above, is important to identify the correct genes as being differentially expressed. However, determining the correct read count numbers is of at least equal importance [33]. We have addressed this issue on two levels by comparing raw counts for the different genes or transcripts among the mapping tools and by comparing differentially expressed genes between plants grown under ambient and cold conditions identified by the different tools.

To investigate the results obtained by the different tools on the basis of raw counts, raw count numbers for each gene/transcript of a single sample from Col-0 and N14 each, generated by the different mappers, were plotted against each other. In general, high similarities among the mappers were observed, indicated by correlation coefficients close to 1. Similarly, when the raw counts were compared between mappers for all 36 biological samples generated in this study, R_v_ values close to 1 indicated a good correspondence in the expression levels computed by all seven software tools.

To analyse the effects of the mapping tools on the DGE analysis, we compared expression levels of control plants grown at ambient temperature with expression levels of plants that were exposed to 4 °C for three days (cold acclimation; compare [17] for a detailed description). Significantly differentially expressed genes were in all cases identified using the *DESeq2* tool. The results showed that the raw counts generated by the different mappers resulted in clear differences in the number of significantly differentially expressed genes, with an overlap between mappers from 98.0% between kallisto and salmon in Col-0, and 92.1% between bwa and STAR in N14. The small sample size (three samples per condition and accession) may of course contribute to the uncertainty in identifying differentially expressed genes unambiguously [34]. However, this sample size is currently the standard in biological experiments and therefore our results give a realistic impression of what the user can expect from the performance of these tools.

Finally, the results from *DESeq2* and from the DGE-pipeline of CLC were compared. Interestingly, CLC identified about 50% more differentially expressed genes than *DESeq2*. Since the same alignments for downstream analysis were used in both cases, this difference cannot originate from differences in the mapping and raw count generation. Therefore, the normalization (to one million counts) as well as the statistical tests used by CLC must have led to these differences. In a transcriptome analysis of mouse tissues, different DGE tools such as *DESeq2* and CLC were compared, resulting in a better performance for *DESeq2* compared to both CLC approaches [35]. The results were experimentally validated by qRT-PCR for 18 differentially expressed genes. For the CLC Baggerly approach large differences to qRT-PCR results were shown. The CLC EDGE approach yielded results that were more similar to the expression changes found by qRT-PCR and those detected by *DESeq2*. However, in our analysis, the CLC approaches yielded results that were largely different from those obtained by *DESeq2*.

## 4. Materials and Methods

### 4.1. Experimental Dataset

RNA samples of the *Arabidopsis thaliana* accessions Col-0 and N14 were used for RNA-Seq as described in detail recently [17]. Plant material was collected from three independent biological experiments resulting in a total of 36 samples. Samples were taken after 28 days of growth at 20 °C, after an additional three days of cold acclimation at 4 °C, after a subsequent seven day period at 20 °C and after a final three days at 4 °C. Additionally, samples from developmental control plants were taken after 35 days at 20 °C and a subsequent three days of cold acclimation at 4 °C (Details of all samples are given in Table A3). Library preparation and sequencing were performed by the Max-Planck Genome Centre Cologne, Germany (https://mpgc.mpipz.mpg.de/home/). Libraries were constructed with NEBNext Ultra Directional RNA Library Prep Kit for Illumina (New England Biolabs) including polyA enrichment. Illumina HiSeq 3000 technology was used for sequencing and yielded 150 base pair (bp) long single end reads. RNA-Seq raw counts are available at GEO [36] under the accession number GSE112225. A detailed biological analysis of the RNA-Seq data has been presented recently [17].

### 4.2. Mapping

Quality control of the raw reads and adapter trimming have been described previously [17]. The genomic FASTA sequence, cDNA and GTF annotation files of *Arabidopsis thaliana* Col-0 were downloaded from EnsemblPlants [37], version TAIR10, release 31 [38]. For read mapping bwa, CLC Genomics Workbench, HISAT2, kallisto, RSEM, salmon and STAR were used, employing pre-defined default parameters as far as possible (Table 6). Bwa aln was used for higher sensitivity and resulting sai files were converted into alignment files with bwa sampe. For kallisto and salmon it was necessary to set parameters for single-end data, define the estimated average read length as well as its estimated standard deviation. As index mode for salmon, --type quasi and a stranded library type were chosen. For expression quantification kallisto and salmon were run in quant mode. For STAR, 1-pass mode was used and additional parameters were defined to sort the alignments, to limit multi-mapping and to keep unmapped reads in the alignments as well as generating the gene count output. HISAT2 was run with default parameters, for index generation annotation was included (Table 6). All tools are freely available except the CLC Genomics Workbench which is a commercial tool that requires purchase of a license. For the mappings without annotation, HISAT2 was run with default parameters and without inserting the annotation into index generation. STAR was run in the 2-pass mode. To determine the reads mapping on exons, *featureCounts* v2.0.0 [39] (--primary -T 10 -f -O -F GTF -t exon -g gene_id) was used. Expression values were natively generated by five of the seven mappers. For bwa, samtools idxstat and for HISAT2, *featureCounts* v. 2.0.0 [39] were used to determine raw counts. For mapping statistics and further analysis of the alignment files, samtools v1.3 [40] was employed.

### 4.3. Comparison Based on Expression Values

For the comparison of the expression values (raw counts), samples A for Col-0 and B for N14 (grown under 20 °C control conditions; see Table A3) were chosen as an example. Raw counts were log_2_(counts + 1) transformed and results visualized with the R-package *ggplot2* [42]. For an overall comparison the R_v_ coefficient [43] based on correlation matrices of the unfiltered raw count tables of samples A and B over all mappers was calculated using the R-package *FactoMineR* [44]. Spearman correlation was used for correlation analysis and the significance of the results was tested as described [45]. The results were visualized employing the R-package *corrplot* [46].

### 4.4. Differential Gene Expression

Prior to the differential gene expression (DGE) analysis, estimated read counts provided by RSEM, kallisto and salmon were rounded to obtain integer values. The resulting count tables for all mappers were filtered to discard lowly expressed genes by keeping only those with a sum greater than five counts per gene for all 36 samples. The DGE analysis was performed using the R-Package *DESeq2* [18] including the normalization step. For CLC, alignment files were extracted and processed in the same way as for the other six mappers. Data was loaded with the function DESeqDataSetFromMatrix. Additional parameters for DGE were used as follows: test = “Wald”, fitType=”local” and including a batch effect correction in the design formula. For determining differentially expressed genes, a threshold *p*-value < 0.1 after false-discovery rate correction [47] and an absolute log_2_ fold change > 1 were used. Results of the comparison control vs. cold acclimation (Table A3) for Col-0 (samples A, M, Y vs. C, O, AA) and N14 (samples B, N, Z vs. D, P, AB) were investigated in detail.

Additionally, the built-in CLC workbench plugin for DGE was tested based on the mappings generated by CLC. Data was normalized “By totals” to a value of 1,000,000. Normalized data was used for determination of differentially expressed genes using the “Empirical analysis of DGE” [19] and “Baggerly’s test on proportions” [20] with multiple testing correction of the generated p-values [47]. Next to the control vs. cold acclimation comparisons described above, the cold acclimated developmental controls (samples I, U, AG for Col-0 and J, V, AH for N14) were compared to the second cold stress treatment (samples K, W, AI for Col-0 and L, X, AJ for N14; Table 1). The numbers of significantly differentially expressed genes (FDR *p* < 0.05, abs(log_2_ fold change) > 1) were compared with the results obtained by *DESeq2* based on the STAR alignments.

### 4.5. Mapping of in Silico Generated Reads

To investigate the mapping quality of the tools, reads were generated in silico using the *A. thaliana* transcriptome (TAIR10) and applying a sliding window approach (window size: 150 bp, shift: 1 bp) resulting in approximately 58 × 10^6^ in silico reads. Reads were mapped with HISAT2 (using the same parameters as above), RSEM and STAR (without --outFilterMultimapNmax and --alignSJDBoverhangMin). For identification, the in silico reads contained the transcript name and the position of the read on the transcript as identifiers. Additionally, the GTF annotation file was reduced to the exon entries and the overlap with the resulting alignment files of HISAT2, RSEM and STAR was determined with bedtools [48]. Furthermore, transcript IDs were compared between alignment entry and GTF entry to identify correctly mapped reads.

## 5. Conclusions

All tested mappers provided highly comparable results for mapping Illumina reads from the genetically distinct Arabidopsis accessions Col-0 and N14 to the Col-0 reference genome or transcriptome. The same was true for the determination of DGE when *DESeq2* was used for processing. We conclude that all seven mappers can be equally used for RNA-Seq data analysis in Arabidopsis, even with different accessions. The only caveat is that using the CLC software for the identification of DGE yielded strongly varying results. Further research will be needed to establish whether read mapping to more complex genomes with larger non-coding regions or higher ploidy levels would pose additional challenges that may reveal larger differences between the mappers.

## Figures and Tables

**Table 1:** Number of expressed genes identified in all samples before and after filtering out lowly expressed genes.

	Bwa	CLC	HISAT2	Kallisto	RSEM	Salmon	STAR
Before filtering	32,243	33,602	33,602	32,243	33,602	32,243	33,602
After filtering	24,197	23,903	24,840	24,810	25,144	24,574	24,515

**Table 2:** DGE analysis using the CLC software.

		DESeq2	CLC			
Comparison	Accession		Baggerly	Overlap DESeq2	EDGE	Overlap DESeq2
C28P3/C28	Col-0	2014	3034	1013	2921	1006
	N14	2101	3414	1061	3311	1052
C28P3L7T3/C35P3	Col-0	1	98	0	86	0
	N14	1	168	0	259	0

Differential gene expression was calculated with *DESeq2* (FDR < 0.1, abs (log2FC > 1), based on STAR alignments and two CLC approaches after Baggerly and EDGE (FDR < 0.05, abs (log2FC > 1)).

**Table 3:** Comparison of selected key features of the used mappers. Features indicated by X are included in the specified mapper.

	Bwa	CLC	HISAT2	Kallisto	RSEM	Salmon	STAR
Reference							
Genome		X	X				X
Transcriptome	X	X	X	X	X	X	X
Needs annotation	X	X		X	X	X	
Specifications							
Alignment-based	X	X	X		X		X
Pseudo-alignment				X		X	
Expression values		X		X	X	X	X
Splice aware		X	X				X
Commercial software		X					

**Table 4:** Fraction of reads mapped to known exons for HISAT2 and STAR.

	HISAT2		STAR	
	Col-0	N14	Col-0	N14
Assigned to exon	94.34	90.70	93.05	90.72
Unmapped	1.10	5.16	0.50	1.99
Multimapped	4.01	3.61	5.93	6.77
No Feature (intergenic)	0.55	0.53	0.51	0.53

To test accuracy of HISAT2 and STAR, reads of the 36 biological samples were mapped against the reference genome without including an annotation. More than 90% of reads were mapped for both accessions and mappers to known exons while a small fraction was either unmapped, multimapped or mapped to intergenic positions.

**Table 5:** Mapping of the in silico-generated Col-0 transcriptome using HISAT2, RSEM and STAR.

	HISAT2	in %	RSEM	in %	STAR	in %
**Primary**						
Mapped on right transcript	57,981,570	98.7	58,072,536	98.9	58,000,379	98.8
Mapped on wrong transcript	689,541	1.2	658,699	1.1	668,909	1.1
Unmapped	18,022	0.031	773	0.001	19,526	0.033
Mapped not on known exon	42,875	0.073	0	0.0	43,194	0.1
total reads	58,732,008	100	58,732,008	100	58,732,008	100
**Secondary**						
Mapped on right transcript	962,756	41.5	1,788,234	4.1	727,039	36.9
Mapped on wrong transcript	1,280,622	55.1	42,112,759	95.9	1,164,065	59.1
mapped on different gene	825,766	64.5	38,112,265	90.5	842,864	72.4
mapped on paralog gene	454,178	35.5	3,957,169	9.4	320,812	27.6
mapped on different isoform	678	0.1	43,325	0.1	389	0.0
Mapped not on exon	79,118	3.4	0	0.0	77,647	3.9
total reads	2,322,496	100	43,900,993	100	1,968,751	100

**Table 6:** Overview of the seven mappers used in this study.

Mapper	Version	Parameters	Reference
bwa aln	0.7.13	Default	Li and Durbin (2009) [11]
CLC	9	Default	Qiagen, Hilden, Germany [41]
kallisto quant	0.42.5	--single, -l 150 and -s 25	Bray et al. (2016) [16]
HISAT2	2.1.0	Default	Kim et al. (2019) [19]
RSEM	1.2.30	--bowtie2, --fragment-length-mean 150 & --fragment-length-sd 25	Li and Dewey (2011) [14]
salmon quant	0.6.0	--type quasi, -k 31 --fldMean 150, --fldSD 25 and -l SF	Patro et al. (2017) [15]
STAR	2.5.2a	--outSAMtype BAM SortedByCoordinate --outFilterMultimapNmax 20 --alignSJDBoverhangMin 8 --outSAMunmapped Within --quantMode TranscriptomeSAM GeneCounts	Dobin et al. (2012) [12]

## Abbreviations

RNA-Seq	RNA-sequencing
DGE	Differential Gene Expression
BWT	Burrows–Wheeler-Transformation

## Appendix A

**Table A1:** Number of reads for raw and pre-processed data.

Sample	Number of Reads Raw Data	Number of Reads Pre-Processed Data
A	26,551,078	25,965,205
B	24,160,253	23,723,408
C	24,987,211	24,631,398
D	24,679,891	24,314,564
E	32,902,966	32,265,838
F	25,343,870	24,962,434
G	25,633,391	25,255,295
H	24,767,056	24,276,316
I	22,434,138	22,074,152
J	27,102,013	26,738,311
K	29,909,220	29,473,355
L	30,039,895	29,625,213
M	25,373,173	25,045,811
N	27,401,911	27,059,316
O	32,172,339	31,758,225
P	26,713,325	26,326,809
Q	28,367,001	27,941,198
R	21,784,606	21,476,277
S	23,466,191	23,142,088
T	25,002,989	24,642,826
U	25,470,737	25,137,081
V	32,322,582	31,890,842
W	31,880,034	31,451,153
X	28,614,863	28,223,380
Y	25,396,753	24,312,026
Z	25,402,962	24,351,761
AA	21,934,477	21,095,112
AB	29,068,271	27,924,700
AC	28,363,133	27,205,327
AD	27,538,807	26,446,048
AE	21,048,979	20,198,121
AF	22,915,893	21,786,356
AG	26,195,089	25,161,103
AH	23,710,160	22,348,705
AI	25,915,840	24,936,936
AJ	27,904,776	26,835,785

Pre-processed raw data was filtered for a minimum read length of 80 base pairs and Illumina adapters were removed.

**Table A2:** Number of mapped reads for each mapper and sample.

Sample	bwa	CLC	HISAT2	kallisto	RSEM	salmon	STAR
A	24,990,288	25,070,332	25,727,064	25,202,788	25,068,400	25,488,500	25,877,150
B	22,235,860	22,831,185	22,831,427	22,489,984	22,450,834	22,625,100	23,535,895
C	23,568,631	23,650,969	24,398,527	23,911,331	23,729,822	24,096,400	24,545,823
D	22,665,145	23,292,374	23,392,011	23,182,011	23,001,552	23,294,400	24,114,936
E	31,067,889	31,136,183	31,948,360	31,315,586	31,186,635	31,651,600	32,144,692
F	22,079,186	23,828,249	22,529,274	23,226,055	22,975,469	23,289,400	24,362,368
G	24,360,053	24,368,639	25,003,451	24,630,743	24,435,931	24,818,000	25,152,392
H	22,607,768	23,256,060	23,230,510	22,983,234	22,847,497	23,135,800	23,972,434
I	20,887,128	20,897,575	21,647,744	21,094,905	21,052,741	21,301,500	21,759,724
J	25,002,889	25,748,821	25,729,980	25,530,361	25,258,258	25,626,800	26,525,228
K	28,251,892	28,394,902	29,083,561	28,728,018	28,398,031	28,924,400	29,340,134
L	27,691,133	28,565,611	28,456,640	28,333,833	27,965,330	28,411,700	29,380,081
M	24,027,754	24,158,404	24,771,967	24,370,150	24,159,388	24,539,200	24,947,419
N	25,448,518	26,128,347	26,116,046	25,859,912	25,708,968	25,908,500	26,872,750
O	30,483,322	30,538,741	31,426,082	30,970,549	30,650,488	31,145,900	31,631,436
P	23,748,275	24,471,940	24,562,932	24,318,422	24,070,551	24,406,800	25,332,412
Q	26,863,089	26,968,681	27,679,891	27,157,401	26,977,076	27,405,000	27,843,106
R	19,700,000	20,245,101	20,218,359	19,970,836	19,918,383	20,052,800	20,826,196
S	22,171,458	22,274,280	22,902,423	22,444,868	22,280,936	22,624,300	23,035,647
T	23,165,182	23,815,751	23,748,284	23,543,789	23,398,523	23,638,100	24,456,937
U	24,145,575	24,192,319	24,905,595	24,499,411	24,279,099	24,667,800	25,057,610
V	29,305,198	30,181,899	30,105,423	29,951,050	29,635,355	30,012,700	31,037,834
W	30,171,991	30,240,229	31,135,272	30,619,320	30,314,724	30,820,900	31,321,391
X	26,417,781	27,215,579	27,146,639	26,999,068	26,701,971	27,089,600	28,004,846
Y	23,467,493	23,523,457	24,062,637	23,713,957	23,548,791	23,915,800	24,211,437
Z	22,939,890	23,531,637	23,488,307	23,241,258	23,186,262	23,333,700	24,169,828
AA	20,347,062	20,333,841	20,891,798	20,594,460	20,425,031	20,742,500	21,011,777
AB	26,183,324	26,842,810	26,903,033	26,663,997	26,539,902	26,769,800	27,709,817
AC	26,065,885	26,102,795	26,890,847	26,358,644	26,235,209	26,562,400	27,054,414
AD	24,904,560	25,532,006	25,483,657	25,267,366	25,201,022	25,348,100	26,234,545
AE	19,055,414	19,320,692	19,842,597	19,566,392	19,295,597	19,670,300	19,967,391
AF	17,469,949	18,053,590	18,090,815	17,854,059	17,755,997	17,898,700	18,672,118
AG	24,163,365	24,161,812	24,876,952	24,468,971	24,283,108	24,682,500	25,047,179
AH	20,953,174	21,498,746	21,436,520	21,310,760	21,215,866	21,379,400	22,109,670
AI	23,823,058	23,916,429	24,617,944	24,223,766	23,973,245	24,383,700	24,792,766
AJ	25,023,005	25,804,958	25,767,495	25,481,098	25,325,866	25,563,800	26,594,060
Col-0 %	95.9	96.2	98.9	97.2	96.4	97.9	99.5
N14 %	92.4	95.2	94.9	94.2	93.6	94.6	98.1
**Total %**	**94.1**	**95.7**	**96.9**	**95.7**	**95.0**	**96.3**	**98.8**

Tools are sorted alphabetically by name. Total describes the fraction of mapped reads for both accessions Col-0 and N14.

**Table A3:** Sample list with sample name, condition (Cond.) and accession (Acc.).

Experiment 1			Experiment 2			Experiment 3	
Cond.	Acc.	Sample	Cond.	Acc.	Sample	Cond.	Acc.
C28	Col-0	M	C28	Col-0	Y	C28	Col-0
C28	N14	N	C28	N14	Z	C28	N14
C28P3	Col-0	O	C28P3	Col-0	AA	C28P3	Col-0
C28P3	N14	P	C28P3	N14	AB	C28P3	N14
C35	Col-0	Q	C35	Col-0	AC	C35	Col-0
C35	N14	R	C35	N14	AD	C35	N14
C28P3L7	Col-0	S	C28P3L7	Col-0	AE	C28P3L7	Col-0
C28P3L7	N14	T	C28P3L7	N14	AF	C28P3L7	N14
C35P3	Col-0	U	C35P3	Col-0	AG	C35P3	Col-0
C35P3	N14	V	C35P3	N14	AH	C35P3	N14
C28P3L7T3	Col-0	W	C28P3L7T3	Col-0	AI	C28P3L7T3	Col-0
C28P3L7T3	N14	X	C28P3L7T3	N14	AJ	C28P3L7T3	N14

Samples were taken from three independent biological experiments. C28/C35: Control plants after 28 days or 35 days of growth at 20 °C; C28P3/C35P3: plants after an additional 3 days of cold treatment at 4 °C; C28P3L7: cold treated plants after a further 7 days at 20 °C; C28P3L7T3: plants after an additional 3 days at 4 °C.
